# Regulatory T cell transdifferentiation as a driver of obesity and diabetes

**DOI:** 10.3389/fimmu.2025.1698285

**Published:** 2025-11-19

**Authors:** Acelya Yilmazer, Dimitra Maria Zevla, Karsten Kretschmer

**Affiliations:** Molecular and Cellular Immunology/Immune Regulation, Center for Regenerative Therapies Dresden (CRTD), Center for Molecular and Cellular Bioengineering (CMCB), Technische Universität Dresden, Dresden, Germany

**Keywords:** Foxp3, pTreg/tTreg, adipose tissue, pancreas, β cells, metabolism, obesity, autoimmunity

## Abstract

Foxp3^+^ regulatory T (Treg) cells exhibit remarkable plasticity, enabling them to phenotypically and functionally adapt to diverse immune responses across tissues. However, this plasticity comes with the risk of lineage instability, including downregulation of Foxp3 and acquisition of pro-inflammatory effector programs. Although Treg transdifferentiation has been implicated in autoimmunity, its precise contribution to disease pathogenesis has remained incompletely understood. Recent advances in single-cell RNA and TCR sequencing provide evidence that, in visceral adipose tissue (VAT), the loss of Treg cells during obesity is driven by the selective transdifferentiation of thymus-derived Treg cells in response to local inflammatory stress. We propose that this process fuels chronic inflammation and may represent one pathway linking Treg instability to chronic VAT inflammation and metabolic dysfunction. Here, we summarize emerging evidence for Treg destabilization in VAT and discuss how local inflammatory and systemic metabolic cues may interact to drive this process, drawing conceptual parallels with autoimmune diseases, particularly type 1 diabetes.

## Introduction

Regulatory T (Treg) cells, defined by expression of the lineage specification factor Foxp3, are essential for maintaining immune tolerance and tissue homeostasis (1, 2). To fulfill their specialized roles across a broad range of tissue niches and immunological contexts, Treg cells exhibit remarkable plasticity, enabling them to adapt to local cues and regulate distinct types of immune responses. This plasticity encompasses both phenotypic and metabolic reprogramming, which supports their survival and function in non-lymphoid environments such as adipose tissue (3, 4), skin (5), gut (6), and central nervous system (7, 8). Tissue adaptation is accompanied by the upregulation of context-specific markers, transcription factors, and cytokine receptors. In addition to tissue adaptation, Treg cells can also tailor their suppressive programs to control qualitatively distinct immune responses by acquiring features of conventional T helper subsets (*e.g.*, Th1-, Th2-, and Th17-like Treg cells) (9, 10). For example, Treg cells can express T-bet to suppress Th1 responses, thereby mirroring conventional Th cell polarization states, while maintaining Foxp3 expression (11, 12). This specialization enhances their ability to functionally restrain context-specific inflammation. However, the same plasticity that enables Treg cells to adapt across diverse contexts may compromise their lineage stability, particularly under conditions of chronic inflammation, metabolic stress, or both. In such environments, Treg cells may downregulate Foxp3, lose their regulatory function, and – under certain conditions – even convert into pro-inflammatory, effector-like T cells, a process termed transdifferentiation (13). These events may compromise immune regulation and actively contribute to disease progression, as implicated in autoimmune disorders (14–17) and in metabolically stressed tissues such as visceral adipose tissue (VAT) during obesity (18, 19). In fact, while VAT Treg cells are generally considered protective in maintaining metabolic homeostasis, emerging evidence indicates that their impact is context-dependent and may even become maladaptive under certain conditions, such as aging or elevated IL-10 production (20, 21), with further modulation by sex-dependent differences in Treg function (22, 23).

Understanding the molecular and environmental cues that govern Treg stability versus instability remains a critical area of investigation, with major implications for immune tolerance, disease pathogenesis, and Treg-targeted therapies. Among Treg developmental subsets, peripherally induced Treg (pTreg) cells have been the focus of intense scrutiny, as they are thought to be particularly vulnerable to losing Foxp3 expression due to their dependence on post-thymic acquisition of a stable epigenetic landscape - most notably, demethylation of the conserved noncoding sequence 2 (CNS2) within the *Foxp3* locus (24). Nevertheless, *in vivo* studies revealed that *de novo*–generated Foxp3^+^ pTreg cells can undergo efficient extrathymic CNS2 demethylation, and retain a stable Foxp3^+^ suppressor phenotype even under inflammatory conditions (25). While Foxp3 instability can affect both pTreg and thymus-derived Treg (tTreg) cells (26), its consequences may be especially deleterious in the tTreg subset, which is selected in the thymus for self-antigen recognition, rendering Foxp3 loss a potential source of autoreactive CD4^+^ effector T cells. Emerging evidence suggests that tTreg instability is not merely a hypothetical concept, but may actively contribute to the pathogenesis of pancreatic β cell autoimmunity and obesity-associated VAT inflammation.

## Functional dichotomy of developmental Treg subsets in VAT

**Figure 1:**
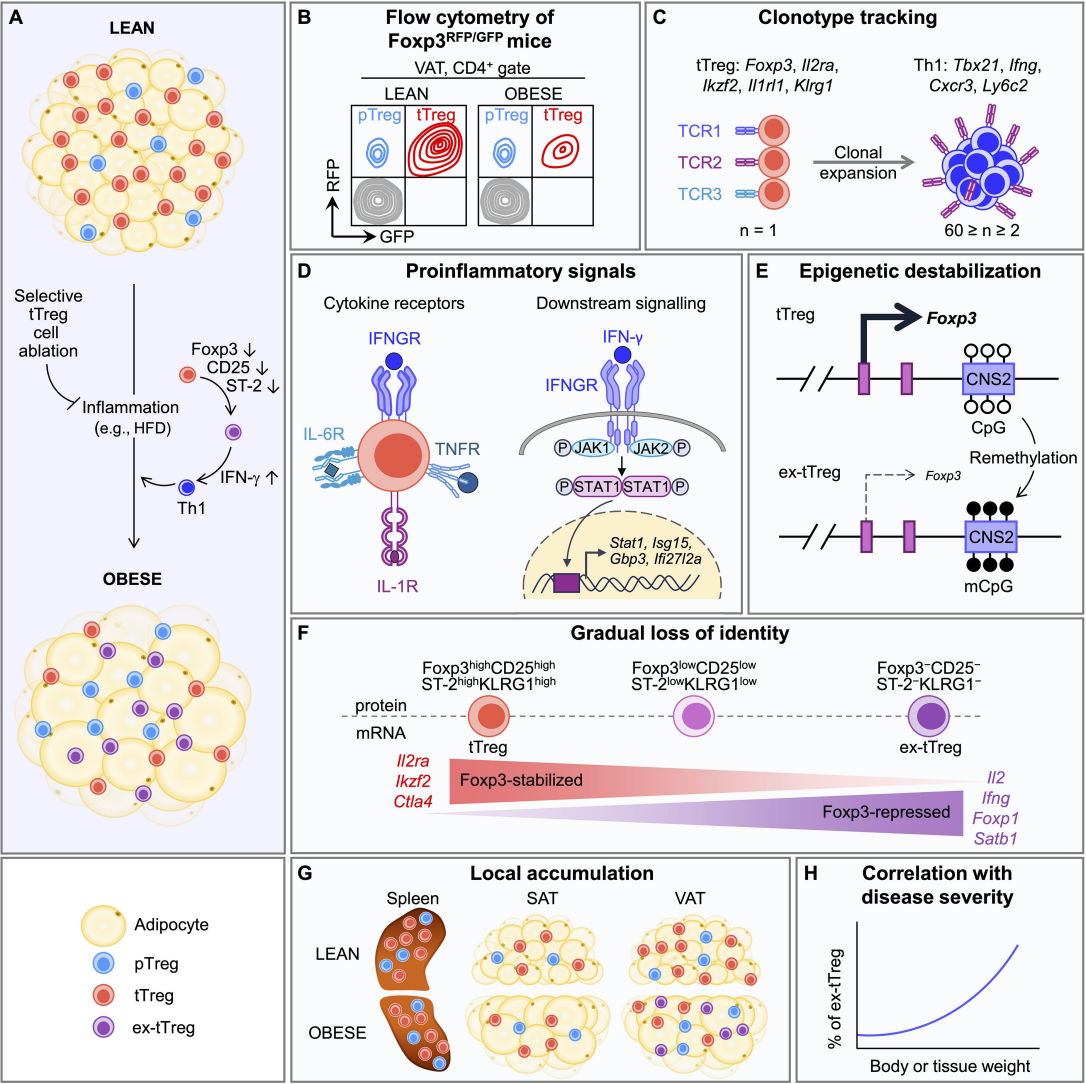
Proposed model of selective tTreg instability as a driver adipose tissue inflammation. **(A)** Treg cells in visceral adipose tissue (VAT) arise from thymic (tTreg) and peripheral (pTreg) lineages. Selective destabilization of tTreg cells causes their conversion into IFN-γ–producing Th1-like effectors that drive inflammation and metabolic dysfunction. Note that genetic tTreg deficiency reduces VAT inflammation, improves steady-state metabolism and prevents diet-induced obesity. **(B-H)** Hallmarks of VAT tTreg transdifferentiation. **(B)** Flow cytometry of Foxp3^RFP/GFP^ mice. The loss of VAT Treg cells associated with obesogenic inflammation is selectively confined to the tTreg subset, while pTreg cells are maintained. **(C)** Clonotype tracking. Unique TCR clonotypes (defined by paired TCR α and β chains) originally recovered from tTreg cells are enriched in T effector compartments exhibiting a Th1 transcriptional signature, while undetectable in naïve T cell and Th2 compartments. **(D)** Proinflammatory signals. Left: Upregulation of cytokine receptors (IL-6, IL-1, TNF, IFN-γ) in VAT tTreg cells. Right: Downstream signaling disrupts lineage stability by altering intracellular pathways. **(E)** Epigenetic destabilization. CNS2 demethylation ensures stable *Foxp3* expression, whereas remethylation undermines stability and promotes Treg transdifferentiation. **(F)** Gradual loss of identity. *Foxp3* mRNA^low^ Treg cells exhibit dysregulated Treg signatures and effector-like programs, reflecting their conversion from stable tTreg to pathogenic T effector cells. **(G)** Local accumulation. Obesity-mediated inflammation drives the selective accumulation of ex-tTreg cells in visceral adipose tissue (VAT), but not in lymphoid organs (*e.g.*, spleen) or subcutaneous adipose tissue (SAT). **(H)** Correlation with disease severity. Abundance of ex-tTreg cells correlates with clinical signs of obesity, including body and adipose tissue weight.

The loss of Foxp3^+^ Treg cells and chronic inflammation in VAT are well-established hallmarks of insulin resistance and obesity pathogenesis (3, 27). To dissect if and how pTreg and tTreg cells are affected, we revisited the role of Treg cells in VAT of dual Foxp3^RFP/GFP^ reporter mice (28) and their derivatives selectively deficient in either the tTreg or pTreg subset (29, 30). Under physiological conditions, Treg cell composition in the lean VAT resembles that of other tissues, with pTreg cells constituting 20–30% and tTreg cells 70–80% of the local Treg compartment (**Figure 1A**). In obesity, this balance appears disrupted, with a selective reduction in tTreg cells, whereas pTreg abundance remains comparatively stable (18).

Strikingly, genetic ablation of pTreg cells led to spontaneous weight gain and adiposity in both male and female mice, even under physiological caloric intake, thereby eliminating the male sex bias typically observed in high-fat diet (HFD)–induced obesity (18). Upon HFD feeding, pTreg-deficient mice developed a particularly severe metabolic phenotype, characterized by enhanced VAT inflammation, hepatic steatosis, and worsened insulin and glucose resistance. Interestingly, their vacated VAT niche was not compensated by tTreg cells in mice with selective pTreg paucity, underscoring a non-redundant role of pTreg cells in maintaining metabolic homeostasis.

In contrast, genetic tTreg ablation mitigated HFD-induced obesity (**Figure 1A**). This was associated with attenuated weight gain, reduced adipose tissue mass, and decreased pro-inflammatory type 1 immunity, accompanied by improvements in systemic metabolic parameters (18). Mechanistically, selective tTreg deficiency was compensated by an expanded Foxp3^+^ pTreg compartment exhibiting a PPAR-γ^+^ST2^+^KLRG1^+^ tissue-adapted phenotype and enhanced expression of genes characteristic of the canonical VAT Treg signature (18). Remarkably, tTreg deficiency conferred improved glucose tolerance and insulin sensitivity even under a normocaloric vegetarian diet (18).

Collectively, these findings suggest that the loss of VAT Treg cells in obesity – particularly those of thymic origin – may contribute to, rather than merely reflect, exacerbated metabolic inflammation. Below, we discuss emerging mechanistic insights into how VAT inflammation may influence Treg cell fate decisions and outline testable hypotheses regarding the potential role of selective tTreg transdifferentiation as a pathogenic driver in obesity.

## Transdifferentiation of Treg cells into pathogenic T effector cells

In general, Treg stability is governed by extrinsic factors such as inflammatory cytokines (14, 31, 32), metabolic stress (10), and tissue-specific cues (33), with the loss of sustained Foxp3 expression and epigenetic integrity predisposing them to the acquisition of pro-inflammatory effector-like phenotypes (14, 15, 17, 31, 32). Although Treg transdifferentiation into pathogenic CD4^+^ effector cells has been implicated in the pathogenesis of several autoimmune diseases in murine models (14, 15, 17), its physiological relevance *in vivo* remains difficult to establish conclusively, largely due to technical limitations and the typically low abundance of such cells. In fact, Foxp3^+^ Treg cells are remarkably stable under homeostatic conditions and across diverse inflammatory contexts, including IL-2 deprivation (34). Studies conducted in lymphopenic environments demonstrate that Treg instability is largely confined to a minor subset of recent thymic emigrant naïve Treg cells (26), underscoring the overall lineage fidelity of mature Treg cells.

Evidence for Foxp3 loss and lineage instability *in vivo* has primarily come from genetic fate-mapping models, such as Foxp3-Cre × Rosa26-YFP mice, in which Foxp3-driven Cre recombinase irreversibly activates a fluorescent reporter (*e.g.*, YFP), permanently marking cells irrespective of their subsequent Foxp3 expression status. However, interpretation of YFP^+^Foxp3^-^ cells in peripheral tissues must be approached with caution, as such cells may not solely reflect peripheral Foxp3 downregulation, but could also result from abortive Treg lineage commitment during thymic development (34, 35). Despite these caveats, several studies have reported the spatial accumulation of YFP^+^Foxp3^-^ cells in inflamed tissues (14, 15, 17), along with their ability to produce pro-inflammatory cytokines upon *in vitro* restimulation (14, 15, 17). These findings have been interpreted as indicative of a pathogenic role in disease progression. Still, direct evidence that ex-Treg cells acquire effector functions sufficient to drive pathology remains limited, largely because their low frequency hampers functional assays.

Recent advances in single-cell transcriptomics have begun to shed light on the cellular trajectories and molecular events associated with Treg destabilization in highly inflammatory environments, such as VAT. These studies provide important mechanistic clues as to how VAT inflammation may influence Treg cell fate decisions (18), consistent with the emerging hypothesis that selective tTreg transdifferentiation contributes to obesity-associated inflammation (**Figure 1A**).

## tTreg transdifferentiation as a driver of VAT inflammation

### Selective loss of VAT tTreg cells

In the lean state, the VAT CD4 T cell compartment harbors an abundant population of Foxp3^+^ Treg cells, comprising 40-60% of CD4^+^ T cells, and characterized by co-expression of the transcription factor PPAR-γ and the IL-33 receptor ST2. These tissue-adapted Treg cells play a pivotal role in maintaining local homeostasis by suppressing pro-inflammatory type 1 responses (3, 4) and regulating adipogenesis (36). The onset of obesity, however, triggers a shift in the VAT from an anti-inflammatory type 2 to a pro-inflammatory type 1 environment (27), accompanied by a marked reduction in the Foxp3^+^ Treg cell compartment (3). Our preliminary data from longitudinal multi-color flow cytometry in HFD–fed Foxp3^RFP/GFP^ dual-reporter mice indicated a progressive decline in Foxp3^+^ Treg cells within the VAT CD4^+^ T cell population – a phenomenon not observed in subcutaneous adipose tissue or lymphoid tissues (18). This decrease was primarily accounted for by a reduction in RFP^+^GFP^+^ tTreg cells, while the RFP^+^GFP^-^ pTreg compartment remained largely stable (**Figure 1B**). These observations point to a selective vulnerability of tTreg cells under obesogenic conditions, without evidence for compensatory expansion of pTreg cells within the vacated niche.

### TCR-based lineage tracing

Single-cell RNA sequencing combined with paired TCR sequencing (scRNA/TCR-seq) has indicated that VAT Treg cells represent a distinct, tissue-adapted population shaped by local antigenic and environmental cues (37). These cells exhibit clear evidence of clonal expansion, indicative of antigen-driven selection and local proliferation within the VAT (38). Their TCR repertoire is largely non-overlapping with that of Treg cells in secondary lymphoid organs, suggesting that VAT Treg cells primarily respond to tissue-restricted antigens. As expected, considering their reduction in abundance, obesity has also been shown to alter the composition of the VAT Treg TCR repertoire (39).

To assess VAT Treg stability and plasticity, we performed scRNA/TCR-seq of CD4^+^ T cells from obese Foxp3^RFP/GFP^ mice (18). VAT Treg cells formed multiple microclones, while maintaining substantial diversity across individual mice. Notably, several clonotypes unique to the Treg cluster were also detected among pro-inflammatory Th1-like non-Treg cells but were absent from naïve and anti-inflammatory type 2 clusters (**Figure 1C**). These shared clonotypes were markedly reduced in HFD-fed mice following genetic tTreg ablation, which coincided with attenuation of obesity-associated pathology, consistent with true transdifferentiation. Together, these findings suggest that, in addition to longitudinal tracking, cross-sectional clonotype overlap may serve as an indicator of recent or ongoing tTreg instability within VAT.

### Pro-inflammatory signals

While IL-2 receptor (IL-2R) signaling is essential for Treg maintenance (40–42), a hallmark of inflammatory microenvironments is limited IL-2 availability due to sequestration and consumption by competing cells. Consistently, VAT tTreg cells in HFD-fed mice exhibited reduced expression of CD25 (18), the high-affinity IL-2R α-chain. IL-2 deprivation, together with IL-2R downregulation, is thought to increase Treg susceptibility to destabilization and transdifferentiation (15, 43, 44), a process that is rare under homeostatic conditions, but becomes pronounced under chronic, inflammatory cytokine-driven stress. In obese VAT, this vulnerability is further compounded by impaired IL-33/ST2 signaling, a critical survival pathway for tissue-resident Treg cells (38, 45). In our study, VAT tTreg cells from HFD-fed Foxp3^RFP/GFP^ mice selectively downregulated ST2 (18), implicating the IL-33–ST2 axis to Treg instability in obesity. More broadly, IL-33 signaling has been shown to support Treg maintenance and suppressive function, as genetic ST2 deficiency predisposes Treg cells to Foxp3 instability and acquisition of a Th17-like phenotype at mucosal sites (31). Although not previously implicated in Treg stability in metabolic tissues, exogenous IL-33 has been shown to restore VAT Treg maintenance (46), an effect counteracted by pro-inflammatory cytokines such as IFN-α, TNF-α, and IFN-γ (47). Consistent with this, VAT tTreg cells in obesity selectively express multiple pro-inflammatory cytokine receptors, including those for IL-6, IL-1, TNF, and IFN-γ (18) – cytokines that have been implicated in promoting Treg instability through altered intracellular signaling pathways (14, 31, 48, 49) (**Figure 1D**, left). Pathway analysis further supported the functional relevance of this profile: for example, elevated IFN-γ receptor expression levels correlated with increased transcription of downstream target genes (**Figure 1D**, right). While we emphasize local inflammatory cues within VAT as key drivers of tTreg transdifferentiation, systemic metabolic signals also modulate tTreg stability in obesity. Elevated circulating levels of leptin and insulin – both tightly correlated with adiposity (50, 51) – directly influence T-cell and Treg function through PI3K–Akt–mTOR, JAK/STAT, and NF-κB signaling pathways (51–57). In this context, chronic hyperleptinemia and hyperinsulinemia may act synergistically with local IL-2 deprivation to destabilize tTreg cells, potentially via epigenetic mechanisms involving the *Foxp3 CNS2* locus and altered STAT5 accessibility (41, 42). Together, these processes highlight how systemic hormonal dysregulation and local cytokine environments cooperate to shape tTreg plasticity and drive immune–metabolic imbalance in obesity.

Collectively, these cytokine and hormonal signals act as central mediators of inflammatory stress, converging to promote effector T-cell polarization and to compromise Treg lineage stability. By disrupting Foxp3 transcriptional and epigenetic regulation, they may foster Treg reprogramming and perpetuate pathogenic immune responses in chronically inflamed metabolic tissues.

### Epigenetic destabilization

*CNS2*, also known as Treg-specific demethylated region (TSDR), plays a key role in stabilizing *Foxp3* expression and maintaining Treg identity (41, 42, 58). During intrathymic Treg development, active *CNS2* demethylation by TET enzymes permits binding of essential transcription factors (including STAT5, Runx1–Cbfβ, CREB, and Foxp3 itself), providing a positive feedback loop that reinforces *Foxp3* expression (24, 25, 41, 42, 58). Interestingly, aberrant TET activity has been implicated in Treg destabilization by promoting *CNS2* methylation at the *Foxp3* locus in the NOD mouse model of type 1 diabetes (T1D) and in children with overt T1D (59). Although *CNS2* remethylation has not yet been directly examined in VAT tTreg cells at the single-cell level, sustained pro-inflammatory cytokine signaling and IL-2 deprivation in obese VAT may disrupt this regulatory axis, leading to *CNS2* remethylation, transcriptional silencing, and ultimately reduced *Foxp3* mRNA expression, preceding a phenotypic shift (**Figure 1E**). Single-cell chromatin accessibility (*e.g*., scATAC-seq) has been widely studied in Treg cells (60–62), whereas single-cell DNA methylation analysis remains challenging but is likely to become feasible with emerging technologies (63, 64).

### Gradual loss of identity

Treg transdifferentiation is not an abrupt binary switch but a gradual, stepwise process marked by the progressive downregulation of Foxp3 and its key stabilizing cofactors, such as Helios and Eos (65, 66). This is accompanied by dysregulation of Foxp3-dependent target genes and a gradual upregulation of context-specific effector programs – including T-bet (Th1), RORγt (Th17), and Bcl6 (Tfh) – reflecting a coordinated shift toward alternative lineage identities. In our single-cell transcriptomic analysis of VAT CD4^+^ T cells, Treg cells were defined based on high expression of *Foxp3* mRNA and canonical signature genes (*e.g.*, *Il1rl1*, *Il2ra*, *Ikzf2*, *Ctla4*, *Klrg1*) (18). We identified a population of CD4^+^ non-Treg cells with low *Foxp3* mRNA and reduced expression of key Treg markers, including *Ikzf2* (Helios), *Il2ra* (CD25), and *Ctla4*, selectively enriched within a Th1-like effector/memory cluster (**Figure 1F**). These *Foxp3*^low^ cells could be further stratified into subclusters showing progressive loss of Foxp3-stabilized genes and concomitant upregulation of Foxp3-repressed targets, including pro-inflammatory cytokines (*e.g.*, *Il2*, *Ifng*) and effector-associated genes linked to VAT inflammation (*e.g.*, *Tnfrsf4*, *Tnfsf8*, *Pdcd1*, *Nr4a3*, *Tigit*). Transcriptional changes closely correlated with protein-level alterations, exemplified by the gradual loss of CD25, ST2, and KLRG1 expression in tTreg cells during obesity progression (see below) (**Figure 1F**). Notably, genetic pTreg ablation was associated with a marked expansion of this subset, suggesting a link between tTreg destabilization and the emergence of pathogenic *Foxp3*^low^ T effector/memory cells.

### Local accumulation

In Foxp3^RFP/GFP^ mice with an additional Cre-activatable Rosa26-YFP reporter, GFP-Cre fusion protein expression in tTreg cells enables correlation of YFP with Foxp3 protein (30). Under steady-state conditions, approximately 10% of YFP^+^ cells lack Foxp3 across tissues, likely reflecting a combination of Treg instability, abortive intrathymic Treg development, and reporter leakiness – underscoring the inherent limitations of genetic Treg lineage tracing. In HFD-fed mice, we observed a pronounced accumulation of YFP^+^Foxp3^-^ cells specifically in VAT, but not in lymphoid organs or subcutaneous adipose tissue (**Figure 1G**). In the obese VAT, up to 60% of YFP^+^ cells lacked Foxp3 (18). Moreover, YFP^+^Foxp3^+^ cells showed reduced expression of key Treg signature proteins, including CD25, CD127, and ST2, with expression levels declining in parallel with Foxp3 downregulation (**Figure 1F**), consistent with compromised Treg identity. This selective enrichment of destabilized and ex-Treg-like cells supports a mechanistic link between HFD-induced VAT inflammation, local proinflammatory cytokine exposure, and tTreg instability with loss of Foxp3 expression *in situ*. Supporting their classification as *bona fide* ex-Treg cells, many YFP^+^Foxp3^-^ cells from obese VAT re-expressed Foxp3 upon *in vitro* re-stimulation (18), a defining feature that distinguishes them from those arising from abortive thymic development (35).

### Correlation with disease severity

A central premise of the hypothesis that tTreg destabilization promotes transdifferentiation into pathogenic effector cells is that disease pathology increases with the number of dedifferentiated cells. As more Treg cells lose stability and acquire pathogenic effector-like functions, they not only fail to suppress inflammation but may actively contribute to it, thereby establishing a self-reinforcing loop in which inflammation promotes Treg instability, which in turn exacerbates immunopathology. Supporting this notion, the pathogenic potential of ex-Treg cells has been studied in autoimmune settings, where they have been shown to secrete pro-inflammatory cytokines upon re-stimulation *in vitro* (14, 15, 17), and to promote EAE upon adoptive transfer (15). Although the low abundance of ex-tTreg cells limits their adoptive transfer when FACS-purified from VAT, we observed that YFP^+^Foxp3^-^ cells accumulating in obese VAT of Foxp3^RFP/GFP^ mice produced IFN-γ (18), a key pro-inflammatory cytokine implicated in metabolic inflammation. Consistent with a pathogenic role, our studies in Foxp3^RFP/GFP^ mice show that HFD feeding consistently induced VAT accumulation of YFP^+^Foxp3^-^ cells, although with inter-individual variability that closely correlates with obesity-related symptoms, including increased body weight and adipose tissue mass (**Figure 1H**), implicating these cells in disease progression. Consistently, selective tTreg paucity was associated with protection from severe HFD-induced obesity in Foxp3^RFP/GFP^ mice (**Figure 1A**) (18). Future studies hold promise for providing more definitive insights into the pathogenic role of ex-tTreg cells. Building on current advances, such investigations could employ transgenic T-cell models expressing prototypic ex-tTreg specificities, together with *in vivo* strategies to modulate their accumulation – either by stabilizing tTreg cells to prevent transdifferentiation or by selectively ablating ex-Treg cells.

## Concluding remarks

Single-cell transcriptomics, combined with dual Foxp3^RFP/GFP^ mice and their derivatives with selective pTreg or tTreg deficiency, has proven useful for dissecting the roles of Treg developmental subsets in adipose tissue inflammation and homeostasis. These studies suggest an unexpected functional dichotomy: pTreg cells act as key regulators of adipose tissue homeostasis, while tTreg cell instability may contribute to inflammatory dysregulation. Future studies are warranted to delineate tissue-autonomous from systemic mechanisms governed by distinct Treg cell lineages, given the complex interorgan cross-talk that integrates adipose tissue, liver, muscle, and central nervous system in the regulation of metabolic homeostasis (7, 67). This goal may be achieved through experimental models enabling tissue-restricted Treg subset ablation or targeted modulation of cytokine receptor signaling pathways.

**Figure 2:**
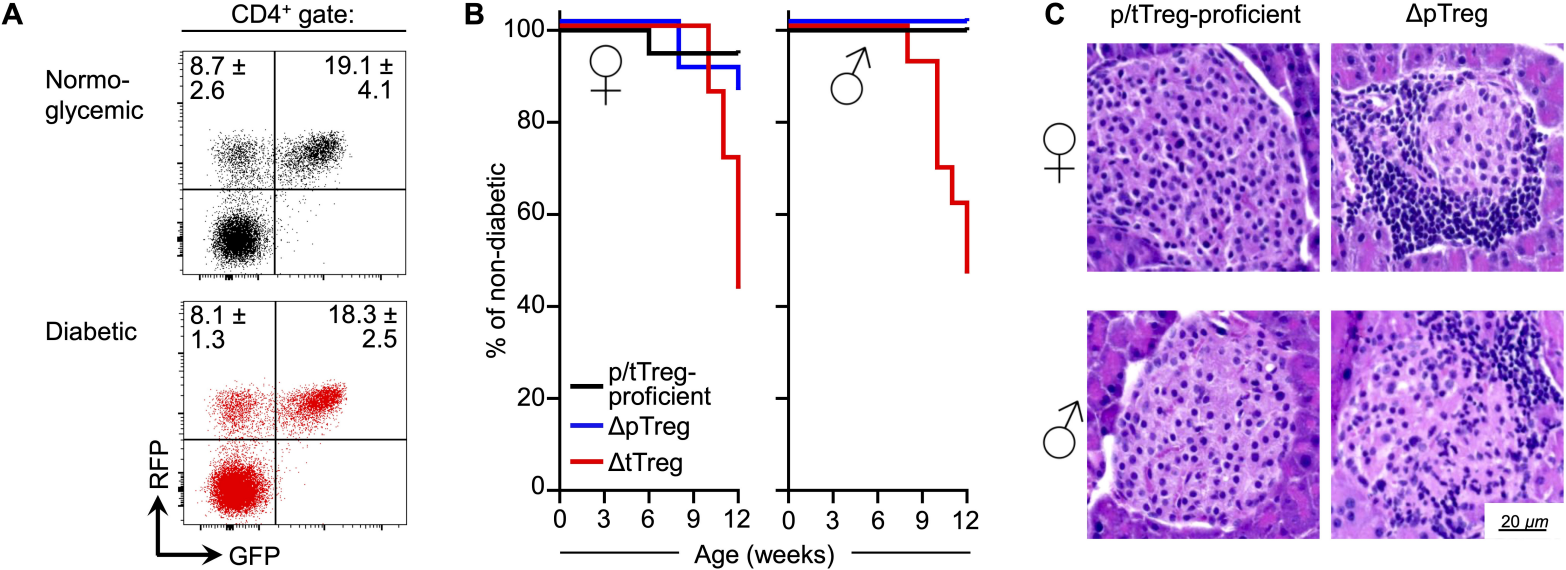
Developmental Treg subsets in the spontaneous NOD mouse model of T1D. **(A)** Representative flow cytometry of dual Foxp3 fluorochrome reporter expression in pancreatic CD4^+^ T cells from normoglycemic (top) and diabetic (bottom) Foxp3^RFP/GFP^ reporter female mice on the NOD background following >14 backcross generations (n = 3 per group). **(B, C)** Mice were backcrossed onto the NOD background for a minimum of four generations. **(B)** Cumulative diabetes incidence in Treg cell-proficient (wild-type), pTreg-deficient (ΔpTreg), and tTreg-deficient (ΔtTreg) mice (n = 12–20 per group). **(C)** Histological assessment of insulitis in Treg cell-proficient (wild-type) and pTreg-deficient (ΔpTreg) mice prior to diabetes onset. Representative H&E-stained pancreatic sections from female (top) and male (bottom) mice illustrates increased leukocytic infiltration in the islets of Langerhans of ΔpTreg mice. Data are adapted from Ref (25).

In contrast, although the critical role of the overall Treg compartment in T1D is well established, the mechanistic contributions of its developmental subsets to constraining pancreatic β cell–autoimmunity remain poorly defined. In the NOD mouse model, Treg instability has been proposed to occur within the autoimmune inflammatory microenvironment of pancreatic islets of Langerhans (17). However, in both non-diabetic and diabetic NOD mice expressing the dual Foxp3^RFP/GFP^ reporter, we failed to detect evidence of selective Treg subset loss (**Figure 2A**), suggesting that the progression to overt diabetes may not be driven by acute pancreatic Foxp3^+^ Treg loss via *in situ* transdifferentiation, as observed in VAT. Instead, Treg cells within the islets appear to largely maintain Foxp3 expression while progressively losing suppressive function – likely reflecting extrinsic influences such as limited IL-2 availability or impaired STAT5 signaling. This distinction underscores divergent mechanisms of Treg dysfunction in metabolic versus autoimmune inflammation, namely lineage destabilization in VAT and functional paralysis in pancreatic islets. Consistently, Treg cells residing within pancreatic islets are thought to become functionally impaired due to limited IL-2 availability (43, 68, 69), which compromises STAT5 signaling, destabilizes Foxp3 expression, and ultimately diminishes their suppressive capacity (40–42). Supporting this, NOD mice with selective tTreg paucity succumb to a particularly severe form of autoimmune diabetes (29), characterized by early onset, high incidence, and a loss of the female bias typically observed in the NOD model (**Figure 2B**). These findings establish tTreg cells as key regulators of β cell autoimmunity, consistent with their central role in maintaining immune tolerance to self (2). In contrast, selective pTreg paucity had only a minor – if any – impact on diabetes incidence (**Figure 2B**), despite exacerbated insulitis observed in both male and female NOD mice (**Figure 2C**).

Nevertheless, destabilization of even a small fraction of Treg cells may suffice to amplify autoimmune responses, particularly when transdifferentiating Treg cells bearing highly pathogenic TCR specificities undergo proliferative expansion within the effector T cell compartment. Yet, the physiological relevance of Foxp3 loss in pancreatic Treg cells during T1D pathogenesis remains to be fully elucidated, as their transcriptional and TCR landscapes have yet to be characterized using single-cell transcriptomic approaches. Looking ahead, studies that integrate scRNA/TCR-seq with chromatin accessibility and DNA methylation profiling will provide a powerful framework to dissect Treg plasticity within both pancreatic and adipose tissue microenvironments. The feasibility of such approaches to capture Treg instability even in human disease has been illustrated by scRNA-seq analyses of peripheral blood from patients with atherosclerosis, which revealed a small cluster of putative ex-Treg cells (70). However, the absence of scTCR-seq precluded direct clonal assessment. Ultimately, defining unstable Treg clones with pathogenic potential may open new avenues for precision therapies in both metabolic and autoimmune diseases.

Overall, multi-omic trajectory analyses hold great promise for uncovering lineage relationships, as well as the transcriptional and epigenetic stability of Treg cells and the clonal architecture of their subsets. Such insights will help clarify the mechanisms underlying pathogenic Treg reprogramming in β cell autoimmunity and adipose tissue inflammation. By defining the molecular determinants of Treg instability, these studies will provide a foundation for targeted therapeutic strategies aimed at preserving immune tolerance and preventing chronic inflammation, ranging from stabilizing Treg cells with low-dose IL-2, IL-2 muteins, or epigenetic modulators, to selectively eliminating unstable clones.

## Data Availability

The original contributions presented in the study are included in the article/supplementary material. Further inquiries can be directed to the corresponding author.
